# Osteopontin’s colocalization with the adhesion molecule CEACAM5 in cytoplasm of carcinoma of tongue and its correlation with the invasion of that diease

**DOI:** 10.1186/1475-2867-12-33

**Published:** 2012-06-27

**Authors:** Fan Zhang, Xu Jin Liu, Xun Qu, Zhen Sheng Hu, Yong Mei Yang, Ling Ma, Pei Liu, Ping Shi, Feng Cai Wei

**Affiliations:** 1 Burns and Plastic Surgery of Qilu hospital of Shandong University, 107#, Wenhua Xi Road, Jinan, Shandong, 250012, People’s Republic of China; 2 Hepatology Department of Qilu hospital of Shandong University, 107#, Wenhua Xi Road, Jinan, Shandong, 250012, People’s Republic of China; 3 Institute of Basic Medical Sciences of Qilu hospital of Shandong University, 107#, Wenhua Xi Road, Jinan, Shandong, 250012, People’s Republic of China; 4 Qilu hospital of Shandong University, 107#, Wenhua Xi Road, Jinan, Shandong, 250012, People’s Republic of China; 5 Oral and Maxillofacial Surgery of Qilu hospital of Shandong University, 107#, Wenhua Xi Road, Jinan, Shandong, 250012, People’s Republic of China

**Keywords:** CEACAM5, OPN, Carcinoma of tongue, Colocalization

## Abstract

The purpose of this study was to investigate the expression of carcinoembryonic antigen-related cell adhesion molecule 5 (CEACAM5) and correlate it with OPN expression and function in squamous carcinoma of tongue.

Paraffin were sections of 80 samples with squamous carcinoma of tongue and 40 samples with normal tissue of tongue for benign lesion having undergone surgery. Immunohistochemistry (IHC) was used to study the distribution of CEACAM5 and OPN, and double–labeling immunohistochemistry was used to observe the relationship between CEACAM5 and OPN expression.

CEACAM5 and OPN are found in normal tissue of tongue, but with different expression pattern. CEACAM5 expression mainly with membranous staining is restricted on the superficial epithelium. However, OPN expression with mainly cytoplasmic staining is restricted on the deep epithelium. No colocalization of CEACAM5 and OPN have been observed in normal tissue of tongue. In squamous carcinoma of tongue, CEACAM5 expression with cytoplasmic staining is different from normal tongue tissue with membranous staining, and the transformation of CEACAM5 distribution from membrane to cytoplasm is an important incident for the invasion and differentiation of tumor. CEACAM5 and OPN are colocalized in cytoplasm, and a significant correlation was observed between the positive colocalization and the negative colocalization in the depth of invasion and the differentiation of the tumor.

## Introduction

Homeostasis in normal tissue is regulated by a balance between proliferative activity and cell loss by apoptosis [1,2]. Attachment to correct extracellular matrix (ECM) is essential for survival and growth of normal adhering cells, whereas cancer cells are able to abrogate this requirement. Several growth factors and cytokines play pivotal roles in the regulation of growth and survival of neoplastic cells through affecting integrin-mediated adhesion to ECM.

Cell adhesion molecules are important mediators of cellular contacts and cellular polarity that also modulate proliferation, differentiation, and invasion. Osteopontin (OPN) is a 70-kDa secreted extracellular matrix glycoprotein with an arginine-glycine aspartate-binding motif capable of interaction with integrin subunits [3-5]. Both OPN and CEACAM5 are important cell adhesion molecules. OPN has been demonstrated to be expressed in a variety of human tissues, including the kidneys, thyroid, gastrointestinal tract, breast, and endometrium, and has been implicated in mediation of cell-cell and cell-extracellular matrix communication that encompass both normal and tumorigenic developmental processes, cell adhesion, spreading, metastasis, and invasion [6-8]. CEACAM5 is a member of the carcinoembryonic antigen and the immunoglobulin superfamily. The CEACAM5 gene, also known as CD66e, codes for the protein, CEA [9]. CEACAM5 was first described in 1965 as a gastrointestinal oncofetal antigen [10], but is now known to be overexpressed in a majority of carcinomas, including those of the gastrointestinal tract, the respiratory and genitourinary systems, and breast cancer [11-15]. Studies have demonstrated that OPN is colocalized with the CEACAM1 and enhances invasion of CEACAM1 expressing cells [16]. At present, no information is available on the expression of CEACAM5 and OPN in squamous carcinoma of tongue. Our study would at evaluating the colocalization of OPN with CEACAM5 and the correlation with the carcinoma of tongue invasion and the degree of differentiation.

## Materials and methods

### Patients

The study included 80 patients with squamous carcinoma of tongue and 40 patients with normal tissue of tongue for benign lesion having undergone primary surgical resection at Qilu Hospital of Shandong University between 2008 and 2010. The clinicopathologic information,including sex, age, tumor stage, and tumor differentiation, was obtained from the clinical records. All the diagnoses were made by three pathologists following the WHO classification of tumors: Pathology and Genetics tumors of digestive system.

### Immunohistochemistry(IHC)

Immunohistochemistry was performed on 4-um-thick routinely processed paraffin sections. CEACAM5 was detected using a mouse monoclonal anti-CEACAM5 antibody (abcam). Sections were dewaxed, and endogenous peroxidase was blocked by immersing the slides in a 3% solution of hydrogen peroxide in methanol for 10 min, followed by antigen retrieval. The slides were immersed in 0.01 mol/L citrate buffer solution (pH 6.0) and placed in a microwave oven for 25 min. After washing in 1 mol/L phosphate-buffer saline (PBS, pH 7.4), the sections were covered with normal serum in a humidity chamber for 30 min at room temperature. Excess serum was rinsed off with 1 mol/L PBS, and the sections were incubated with the primary antibodies in a humidity chamber for 45 min at room temperature. Then sections were rinsed with PBS before incubation with the biotinylated second antibody in a humidity chamber for 40 min at 37°C. After rinsing with PBS, the streptavidin-peroxidase complex reagent (StrepABComplex/HRP DUET, CAKO) was added. Slides were incubated for 45 min at room temperature, then washed in 1 mol/L PBS, and covered with 3,3^,^-diaminobenzidine tetrahydrochloride solution for 15 min under the microscope. Sections were then immersed in running tap water, counterstained with hematoxylin for 1 min, followed by a tap water bath, immersion in a series of alcohol baths of increasing concentrations and xylene, and then covered with coverslips. Negative controls were performed in which the primary antibody was omitted. The slides were performed using a mouse monoclonal anti-OPN antibody (abcam) in the same way as the slides for OPN staining.

### Double-labeling immunohistochemistry

OPN and CEACAM5 were performed by double staining against OPN (abcam) and CEACAM5 (abcam) according to the manufacturer^,^s instructions (Zymed HISTOSTAIN-DS BROAD SPECTRUM). Each antibody labeling was performed as described above. Tween-20 PBS (0.05% TPBS, pH 7.4) was used as the buffer. Staining was performed consecutively with CEACAM5 as the first staining. CEACAM5 and OPN labelings were performed by using different chromogens (AEC AND BCIP/NBT, respectively). Negative controls were performed by displacing primary antibodies with PBS.

### Evaluation of CEACAM5 and OPN staining

Histological and IHC evaluations were independently made by three pathologists. Slides with debating evaluation were re-evaluated, and a consensus was reached. For each sample, at least 3000 carcinoma cells were evaluated for CEACAM5 or OPN expression. We examined the sections at 200× magnification, and the percentage of carcinoma cells with membranous or cytoplasmic staining was determined.

### Statistical analysis

Statistical annlysis was performed by using SPSS 10.0 software package for Windows. The chiaquare test was used to analyze the data. Differences were considered significant at Р<0.05.

## Result

### OPN, CEACAM5 expression and colocalization

**Figure 1:**
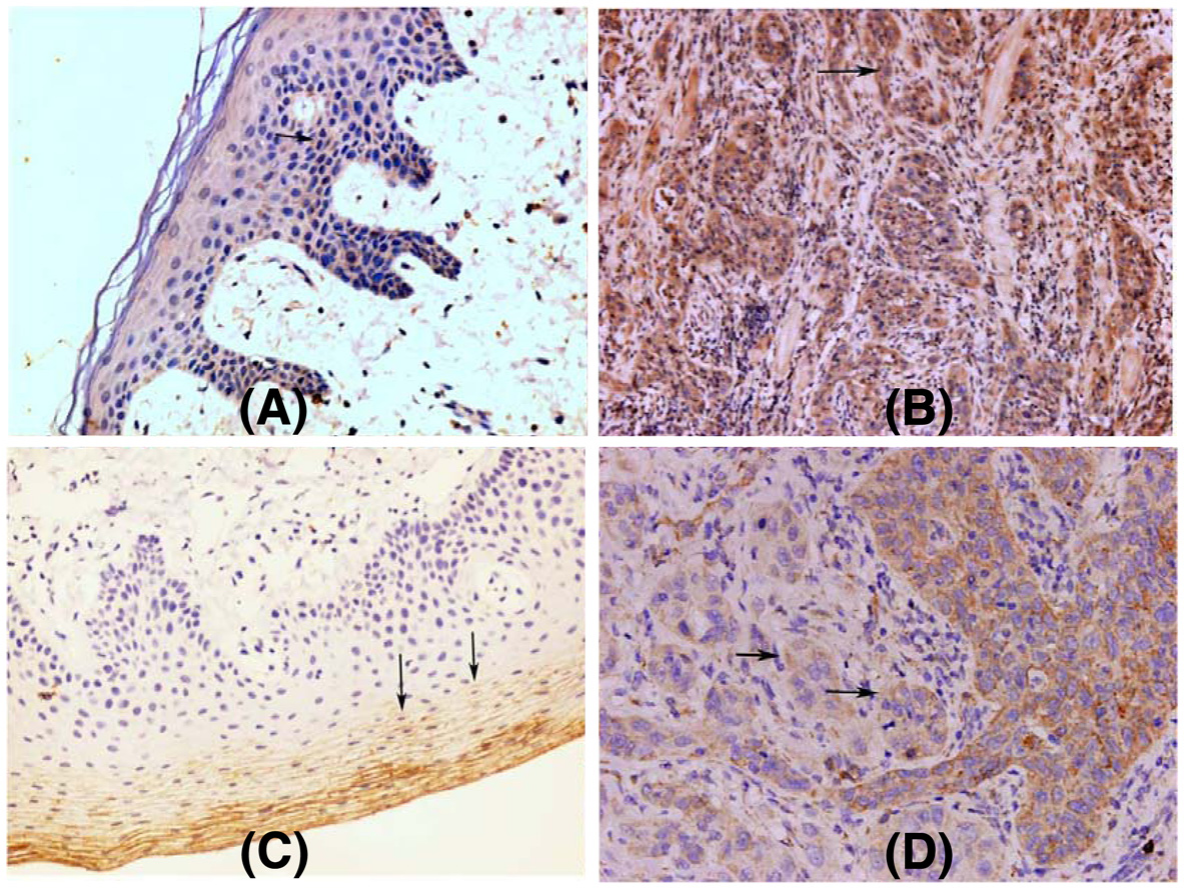
**In both of normal tissue and carcinoma, OPN were all expressed with mainly cytoplasmic staining (arrow) (A and B).** CEACAM5 expression was found in normal tongue tissue and carcinoma with different expression pattern and location. CEACAM5 expression with membranous staining was restricted on the superficial epithelium(arrow) (**C**) in normal tissue. However, CEACAM5 expression with mainly cytoplasmic staining was restricted on the deep epithelium (arrow) (**D**) in squamous carcinoma of tongue.

**Figure 2:**
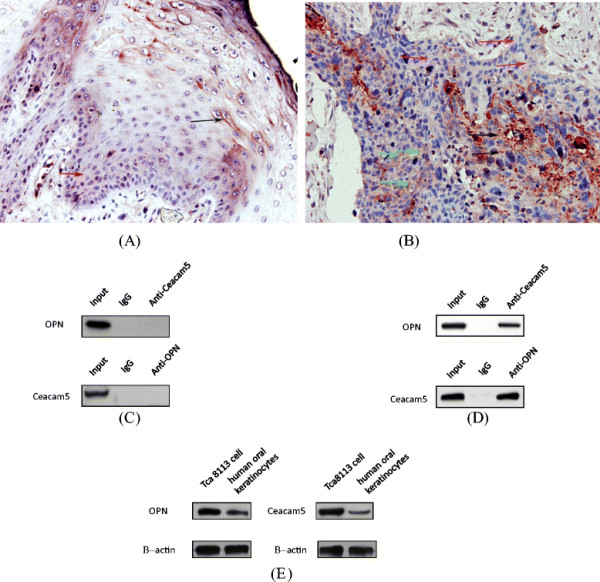
**OPN and CEACAM5 expression with different expression pattern and location were found in normal tongue tissue.** CEACAM5 expression with membranous staining was restricted on the superficial epithelium (black arrow) (**A**), however, OPN expression with mainly cytoplasmic staining was restricted on the deep epithelium (red arrow) (**A**). no colocalization was observation in cytoplasm (**A**). In squamous carcinoma of tongue, OPN (red arrow) and CEACAM5 (black arrow) expression were found with cytoplasmic staining (**B**). CEACAM5 and OPN are being colocalized in cytoplasm (green arrow) (**B**).

Our IHC study showed that OPN and CEACAM5 expression were found in normal tongue tissue, but with different expression pattern and location. CEACAM5 expression with membranous staining was restricted on the superficial epithelium (mainly granular layer, Figure 1A). However, OPN expression with mainly cytoplasmic staining was restricted on the deep epithelium (mainly basal layer, Figure 1B). Fourteen out of all 40 normal tissue CEACAM5 was expressed with membranous staining, six cases with a cytoplasmic staining and five with a mixed pattern of cytoplasmic and membranous staining. Twenty out of all 40 normal tissue expressed OPN with cytoplasmic staining, six cases expressed OPN with membranous staining, seven cases expressed OPN with a mixed pattern of cytoplasmic and membranous staining. The expression patterns showed a significant different between OPN and CEACOM5 in normal tissue (Table 1). According to dual-labeling IHC staining of CEACOM5 and OPN, both of them were expressed in the normal tissue, and no colocalization was observed in cytoplasm (Figure 2A).

**Table 1:** Clinicopathological Characteristics of the Patients

	**Postive samples**	**Staining pattern**			**p values**
		**M**	**C**	**M+C**	
CEACAM5					
Normal	25	14	6	5	
Carcinoma	68	6	56	6	
					<0.05
OPN					
Normal	33	6	20	7	
Carcinoma	70	6	59	5	
					>0.05
**Normal tissue**					
OPN	25	14	6	5	
CEACAM5	33	6	20	7	
					<0.01
**Carcinoma**					
OPN	70	6	56	5	
CEACAM5	68	6	56	6	
					>0.05

**M:** membranous; **C**: cytoblasmic; **M+C:** membranous and cytoblsmic.

Significant, P<0.05.

In squamous carcinoma of tongue, 59 of 80 analyzed tumor samples have been found to express OPN with cytoplasmic staining, six cases with membranous staining and five cases with a mixed pattern of cytoplasmic and membranous staining. 56 of 80 analyzed tumor samples expressed CEACOM5 with cytoplasmic staining, six cases with membranous staining and six cases with a mixed pattern of cytoplasmic and membranous staining in all of 68 analyzed positive tumor samples (Figure 1C and D). CEACAM5 expression with cytoplasmic staining was different from normal tongue tissue with membranous staining. The expression patterns of CEACOM5 showed a significant difference between normal tissue and the squamous carcinoma (Table 1). In addition, we could demonstrate that 42 in all of 80 tumor samples, CEACAM5 and OPN are being colocalized in cytoplasm (Figure 2B) by using double-labeling immunohistochemistry. A significant correlation was observed between the positive colocalization and the negative colocalization in the depth of tumor invasion and differentiation (Table 2). Dual-labeling IHC showed that the movement of CEACAM5 from membrane to cytoplasm may promote OPN expression, and more colocalization cases were detected in squamous carcinoma of tongue. The findings demonstrated that the transformation of CEACAM5 expression pattern and colocalization with OPN might play a critical role in tumor metastasis and invasion.

**Table 2:** Clinicopathologica Characteristics of the Patients

**Variables**	**No.of patients**	**Colocalization**		**p**
		**Negative**	**Positive**	
**Tissue**				
Normal	80	40	0	
Carcinoma	40	42	38	
**Carcinoma**				
Differentiation				
Undifferentiated	43	16	27	
Differentiated	37	28	9	
				<0.05
**Carcinoma**				
Depth of invasion				
T_1-2_	45	29	16	
T_3-4_	35	14	21	
				<0.05

Significant, P<0.05.

## Discussion

The human CEACAM5 protein family encompasses several forms of proteins with different biochemical features. CEACAM5 is an oncofetal glycoprotein, containing 50% carbohydrate with a molecular weight of approximately 200 kDa [17]. CEACAM5 is overexpressed in several tumor types of epithelial origin and is known as an important and extensively used clinical tumor marker for colorectal and other carcinomas [18]. Hence, CEACAM5 is an attractive target for immunotherapeutic purposes because of its expression profile, its role in tumor progression, and its immunogenicity. In benign and malignant lesions of human carcinoma of tongue, the apical membrane expression of CEACAM5 changes to a distinct uniform membrane staining in an early stage of malignant transformation, and the incident of the uniform membrane expression might be due to a loss of or reduction in the interaction among the adhesion molecules with its binding molecules, implicating an important shift towards the malignant phenotype [19]. Using immunohistochemistry, we found that normal tissue of tongue expressed CEACAM5 with apical and uniform membranous patterns, and the carcinomas mainly expressed CEACAM5 with a cytoplasmic pattern. Therefore, membranous distribution of CEACAM5 changed to cytoplasmic staining may indicate a malignant transformation .

OPN is a calcium-binding phosphoprotein with multiple functions. Under physiological conditions, OPN is produced by osteoblasts when stimulated by calcitriol, and it functions by binding to hydroxyapatite to provide the anchoring of osteoclast to the mineral of bone matrix [20]. OPN binds to cells via the vitronectin receptor but also via other integrins and the hyaluronic acid receptor CD44. Several studies have defined OPN as an important glycoprotein with multiple functions and have found it plays a role in basic cellular processes, such as neovascularization and tissue remodeling, which are essential to metastasis and invasion of the tumor [21,22]. Furthermore, several lines of evidence have implicated OPN in angiogenesis, and vascular endothelilal growth factor may induce expression of OPN as well as αv ß3 integrin in endothelial cells [23-25]. Overexpression of OPN in tongue cancers implicated a more aggressive tumor behavior and was an important factor for survival [26,27]. Having assessed the distribution of OPN protein by immunohistochemistry, we found that OPN is expressed in a large percentage of the tissue studied. Significant cytoplasmic OPN staining was observed in a large percentage of all tissue studied.

In the present study, we have investigated the expression pattern of OPN at all tissue studied and its correlation with the expression of CEACAM5. As shown by immunohistochemistry, significant cytoplasmic OPN staining was observed in a large percentage of all tissue studied. CEACAM5 expression with membranous staining was found in normal tongue tissue. In squamous carcinoma of tongue, CEACAM5 expression with cytoplasmic staining was different from normal tongue tissue with membranous staining. According to dual-labeling IHC staining of CEACOM5 and OPN, both of them were expressed in the normal tissue, and no colocalization was observed. However, CEACAM5 and OPN are being colocalized in squamous carcinoma of tongue, and a significant correlation was observed between the positive colocalization and the negative colocalization in the depth of tumor invasion and the differentiation. OPN and CEACAM5 were expressed in both normal and pathological tongue tissues, and their different expression pattern can be potentially useful as an additional diagnostic marker in squamous carcinoma of tongue. Julianne Briese et al. have demonstrated that OPN is colocalized with the CEACAM1 in the extravillous trophoblast of the human placenta and enhances invasion of CEACAM1-expressing placental cells [16]. We have performed a systematic immumohistochemical study on OPN and CEACAM5 protein expression in squamous carcinoma of tongue. Limitations of our study include the absence of in vitro experiment and functional studies. Nevertheless, with the IHC of the correlated expression pattern of OPN and CEACAM5 in carcinoma, our data showed that OPN and CEACAM5 may act as a functional complex in these lesions. This complex may increase invasion and inhibit differentiation of the carcinoma of tongue.

## Competing interests

The authors declare that they have no competing interests.

## Authors’ contributions

FZ and FCW designed the project. All authors performed the experiment. FZ drafted the manuscript. All authors read and approved the final manuscript.
